# Recurrent pericardial syndromes following Boerhaave’s syndrome: a complex clinical presentation and case report

**DOI:** 10.1093/ehjcr/ytaf283

**Published:** 2025-06-14

**Authors:** Musab Eltayeb, Luke Byrne, Gearoid Fitzgerald, Carl Vaughan

**Affiliations:** Cardiology Department, Mercy University Hospital, Granville PI, Cork T12WE28, Ireland; Cardiology Department, Mercy University Hospital, Granville PI, Cork T12WE28, Ireland; Cardiology Department, Mercy University Hospital, Granville PI, Cork T12WE28, Ireland; Cardiology Department, Mercy University Hospital, Granville PI, Cork T12WE28, Ireland

**Keywords:** Boerhaave’s syndrome, Pericardial syndromes, Case report

## Abstract

**Background:**

Recurrent pericardial syndromes secondary to Boerhaave’s syndrome (spontaneous oesophageal rupture) are exceedingly rare and represent diagnostic and therapeutic challenges.

**Case summary:**

We present a case of a 46-year-old male with recurrent pericardial effusions and cardiac tamponade following Boerhaave’s syndrome. Initial management included surgical repair of the oesophageal rupture and medical treatment for the subsequent effusions with colchicine, non-steroidal anti-inflammatory drugs, and corticosteroids. The patient experienced multiple recurrences despite medical therapy, necessitating a multidisciplinary approach. Ultimately, a pericardiectomy was performed, revealing significant pericardial adhesions and inflammation. Post-operative recovery was uneventful with recurrence of effusion.

**Discussion:**

This case underscores the importance of early recognition and multidisciplinary management in recurrent pericardial syndromes associated with oesophageal rupture. While first-line medical therapy can be effective, persistent or refractory cases may require surgical intervention. Early surgical consultation should be considered when medical management fails.

Learning points

- Boerhaave’s syndrome is a rare but severe condition with high morbidity and mortality.
- Recurrent pericarditis following oesophageal perforation poses significant diagnostic and therapeutic challenges.
- Corticosteroids should be considered for refractory pericarditis, but judicious tapering is essential to minimize recurrence risk.
- Early surgical consultation is critical for cases requiring definitive management.

## Introduction

Oesophageal perforation is a rare and potentially life-threatening condition, with iatrogenic causes accounting for ∼45.5% of cases.^1,2^ The condition carries a significant historical mortality rate of up to 50%, though modern interventions have improved outcomes.^3^ Pericardial complications following spontaneous oesophageal rupture are exceedingly rare and sparsely documented in medical literature.^3^ We present a unique case of cardiac tamponade occurring after Boerhaave’s syndrome.

Pericardial disease can arise *de novo* or as a sequela of systemic illnesses. No standardized or licensed therapy specifically targets pericardial effusion or associated syndromes. Pericarditis accounts for ∼0.1% of hospital admissions and 5% of chest pain-related consultations, with a reported recurrence rate of 30%.^4–7^ The aetiology of pericarditis is predominantly idiopathic, though it may also be infectious or non-infectious. Non-infectious causes include autoimmune, neoplastic, metabolic, and traumatic/iatrogenic factors.^5^

Boerhaave’s syndrome, characterized by spontaneous transmural oesophageal rupture due to increased intraluminal pressure, is an uncommon cause of traumatic pericarditis.^3^ We detail the clinical progression of recurrent pericardial effusion and cardiac tamponade following this.

## Summary figure

**Figure:**
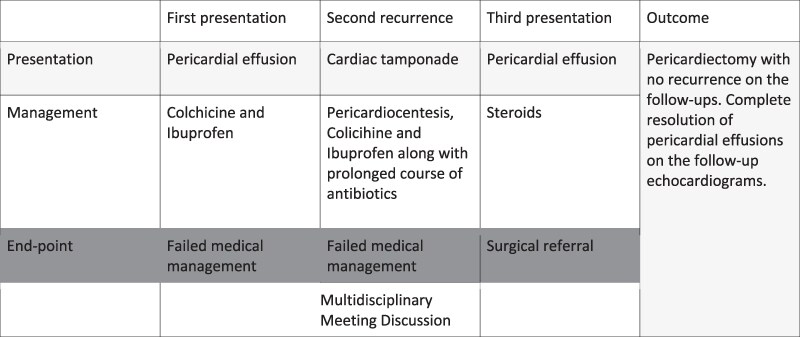


## Case report

### Initial presentation

A 46-year-old male with a history of gastroparesis and stasis oesophagitis presented with severe vomiting, abdominal pain, and right shoulder pain. His alcohol intake was moderate (<14 units per week). Vomitus contained coffee-ground material.

Examination revealed tachycardia (130 beats per minute—b.p.m.), tachypnoea (34/minute), and hypoxia (oxygen saturation SpO2 = 88% on room air). Abdominal tenderness and guarding were noted. Imaging via chest and abdominal X-rays showed pneumomediastinum and bilateral pneumonia more on the left side, raising the suspicion of oesophageal rupture.

**Figure 1:**
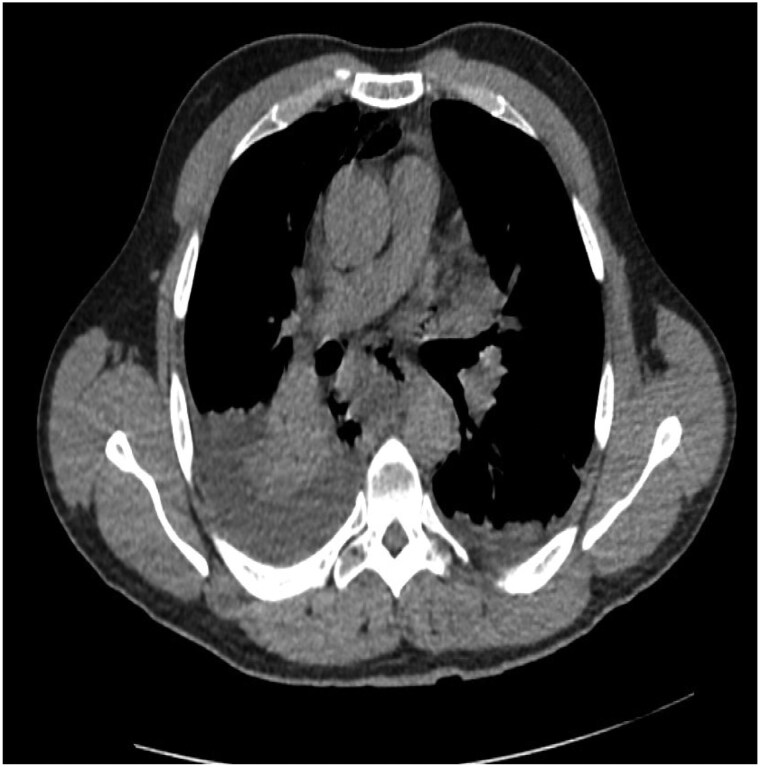
A contrast-free computerized tomography thorax–abdomen–pelvis confirms Boerhaave’s syndrome with pneumomediastinum and bilateral pleural effusions.

A contrast-free computerized tomography thorax–abdomen–pelvis (CT-TAP) confirmed Boerhaave’s syndrome with pneumomediastinum and bilateral pleural effusions (*Figure 1*). Subsequent oesophagogastroduodenoscopy (OGD) revealed a 2 cm right-lateral oesophageal defect, which was repaired surgically. Mediastinal debridement and drainage were performed. Post-operatively, the patient developed acute respiratory distress syndrome, requiring a prolonged intensive care unit stay before discharge.

### First recurrence

**Figure 2:**
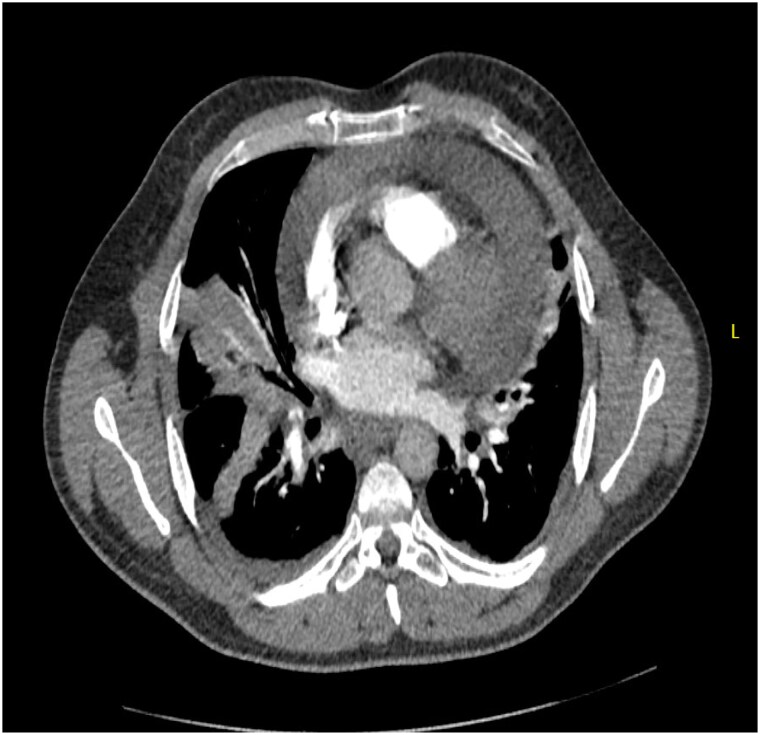
Computerized tomography thorax–abdomen–pelvis showed massive pericardial effusion.

**Figure 3:**
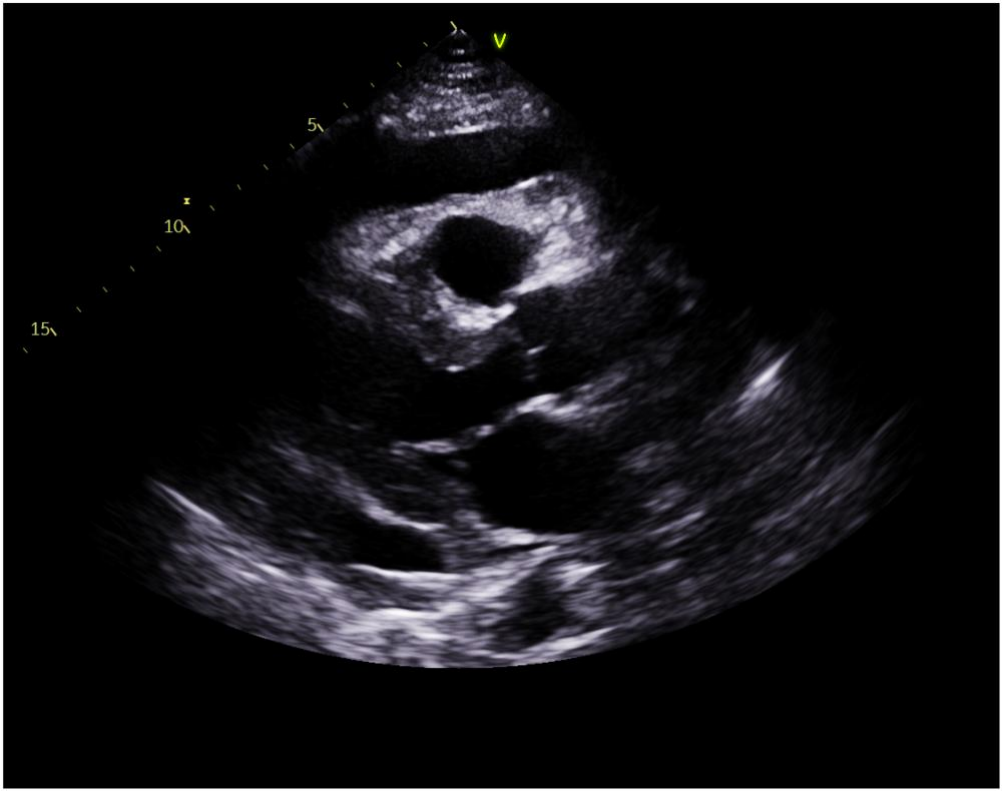
The echocardiogram showed moderate pericardial effusion.

Two months later, the patient presented with left-sided chest pain radiating to the shoulder and dyspnoea. Examination showed tachycardia (112 b.p.m.) and coarse bilateral crepitations. Laboratory findings included elevated inflammatory markers (white cell count WCC 11.2 × 10^9^/L, C-reactive protein 277 mg/L). Imaging via CT-TAP revealed a massive pericardial effusion (*Figure 2*). An echocardiogram confirmed moderate effusion with Doppler variations > 25%, suggesting early tamponade (*Figure 3*). Despite signs of early tamponade, pericardiocentesis was deferred due to a discrepancy between CT-TAP and echocardiographic findings, with the latter only showing moderate effusion without significant haemodynamic compromise.

**Figure 4:**
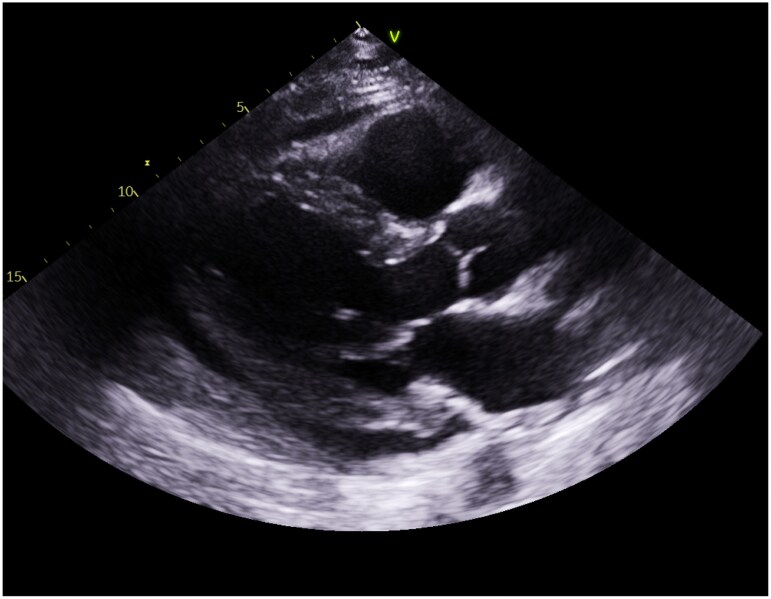
Echocardiogram showing improvement post-initial medical management.

He was treated conservatively with colchicine (500 μg b.i.d.) and ibuprofen (400 mg t.d.s.). A repeat echocardiogram showed significant improvement 3 days later, with only mild residual effusion. The patient was discharged on a three-month colchicine regimen and tapered non-steroidal anti-inflammatory drugs (NSAIDs). Follow-up imaging demonstrated improvement with near-complete resolution (*Figure 4*)

### Second recurrence

**Figure 5:**
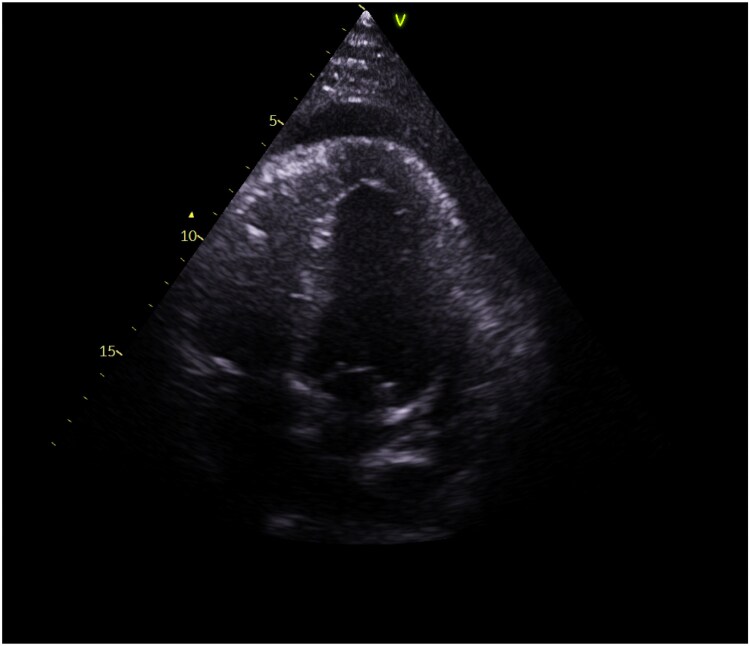
Echocardiogram pre-pericardiocentesis.

Two months post-discharge, the patient returned with dyspnoea and chest pain. Bedside echocardiography revealed severe pericardial effusion consistent with cardiac tamponade (*Figure 5*, Supplementary material online, *Figure S1*). Although he was haemodynamically stable, we performed pericardiocentesis that drained 750 mL of exudative fluid, growing methicillin resistant *Staphylococcus aureus* (MRSA) and *Staphylococcus epidermidis*. Cytology showed acute inflammation only. The patient developed fever, hypoxia, and bilateral pleural effusions. CT imaging and OGD ruled out fistulous communication. Empiric vancomycin was initiated and later adjusted to ertapenem and daptomycin based on microbiology advice for MRSA and *S. epidermidis* coverage. A six-week antibiotic course was completed via a peripherally inserted central catheter (PICC), despite the possibility of contamination.

### Third recurrence and complications

Three weeks post-discharge, the patient presented with PICC line-associated upper limb thrombosis, which was treated as provoked venous thrombosis. The PICC line has been removed, and a direct oral anticoagulant was given for 3 months. Imaging revealed increased pericardial effusion. A cardiac MRI confirmed the recurrence of the effusion (see Supplementary material online, *Figure S2*), prompting corticosteroid initiation (prednisolone 40 mg daily with a slow taper). Repeat imaging showed marked improvement (see Supplementary material online, *Figure S3*). However, symptoms recurred upon steroid tapering, leading to a multidisciplinary consensus for surgical pericardiectomy.

### Surgical intervention and outcome

Pericardiectomy via sternotomy revealed significant adhesions and inflamed visceral pericardium. Histopathology confirmed fibrinous pericarditis with chronic inflammatory infiltrates. Post-operative echocardiography showed complete resolution of effusion with preserved ventricular function. The patient continued on the tapering steroids and colchicine. He remained asymptomatic during subsequent follow-ups, upon completion of the steroids, along with colchicine treatment.

## Discussion

This case highlights the complexity of managing recurrent pericardial syndromes post-Boerhaave’s syndrome. Pericardial effusion likely resulted from direct contamination of the mediastinum with digestive contents. Additional factors, including micro-pericardial laceration during the initial surgical repair, may have predisposed the patient to persistent inflammation with recurrent effusions.^8^

Inflammatory markers were elevated during the first two presentations, which was one of the reasons antibiotics were initiated, despite the growth being considered likely a containment. The recurrence despite antibiotic treatment supports the hypothesis that the condition is likely multifactorial (see Supplementary material online, *Figure S4*).

According to the ESC guidelines,^5^ pericardial syndromes may be caused by infectious (most commonly viral, but also bacterial, fungal, or parasitic) or non-infectious aetiologies. Although cultures showed some growth, contamination is the most likely explanation. Non-infectious causes include autoimmune, neoplastic, traumatic, iatrogenic, and metabolic origins. In this case, the patient likely falls under traumatic/iatrogenic, and possibly metabolic, due to direct contamination following the rupture, given the anatomical proximity of the oesophagus to the mediastinum.

According to ESC guidelines on pericardial diseases, the first-line therapy for acute pericarditis includes colchicine in combination with NSAIDs to reduce the recurrence rates.^5^ In recurrent cases, prolonged colchicine therapy with gradual NSAID tapering is recommended. Although corticosteroids are generally avoided due to higher recurrence risk, they are appropriate in refractory cases.^5,9,10^ Despite initial guideline-directed medical therapy, our patient experienced aggressive recurrences necessitating pericardiocentesis, prolonged antimicrobial therapy, and ultimately, pericardiectomy.^10^ The ESC recommends early surgical consultation in chronic, relapsing pericarditis unresponsive to medical therapy. This case highlights the importance of a tailored approach that integrates medical and surgical strategies for optimal patient outcomes.^11^

## Supplementary Material

ytaf283_Supplementary_Data

## Data Availability

The data underlying this article are available in the article and its online Supplementary material.

## Lead author biography

MBBS, National Ribat University; BST, RCPI.

## Supplementary material

**Consent**: The authors confirm that consent was obtained from the patient to submit and publish the report in accordance with COPE guidance.

**Funding:** None declared.
