# Supplementary material for: Recurrent pericardial syndromes following Boerhaave’s syndrome: a complex clinical presentation and case report
Source: Eur Heart J Case Rep. 2025 Jun 14;9(7):ytaf283. doi: 10.1093/ehjcr/ytaf283 (PMC12233009; doi:10.1093/ehjcr/ytaf283)
Supplement: ytaf283_Supplementary_Data [file ytaf283_supplementary_data.docx]

Supplementary figures:

Figure S1:

Echocardiogram post-pericardiocentesis:
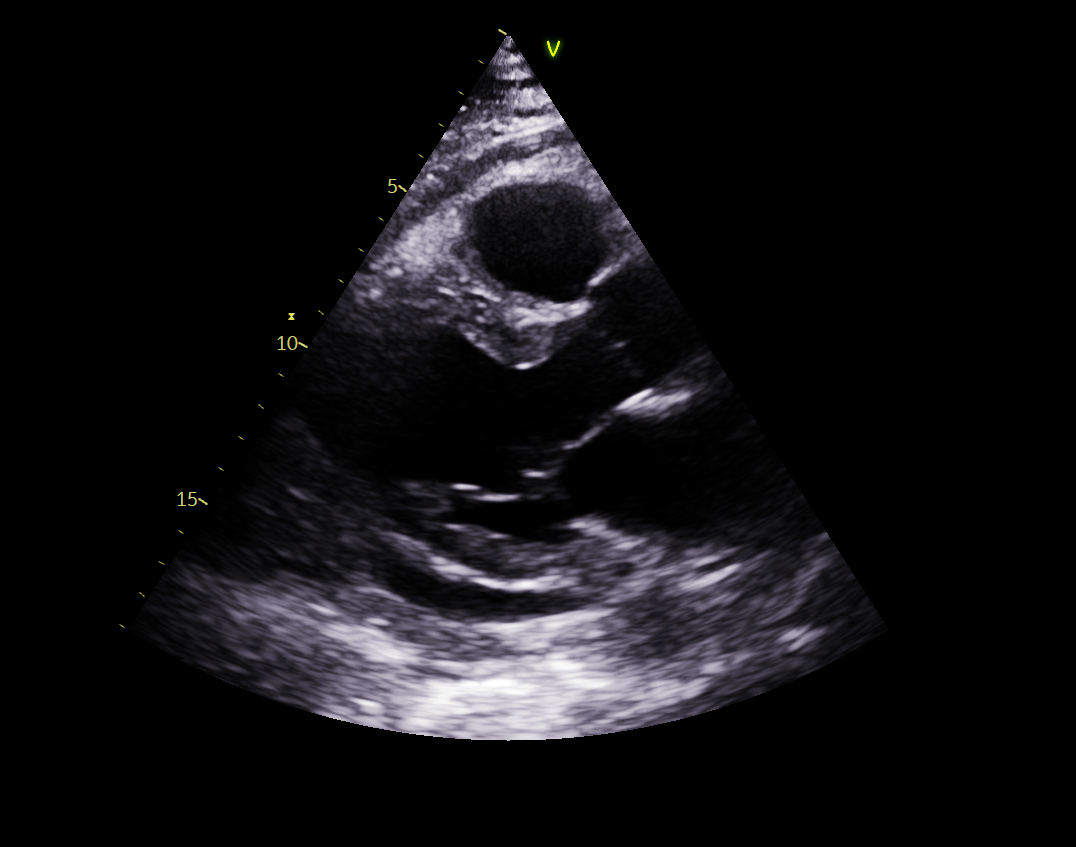


Post-pericardiocentesis

Figure S2:

Cardiac MRI before the steroid treatment:


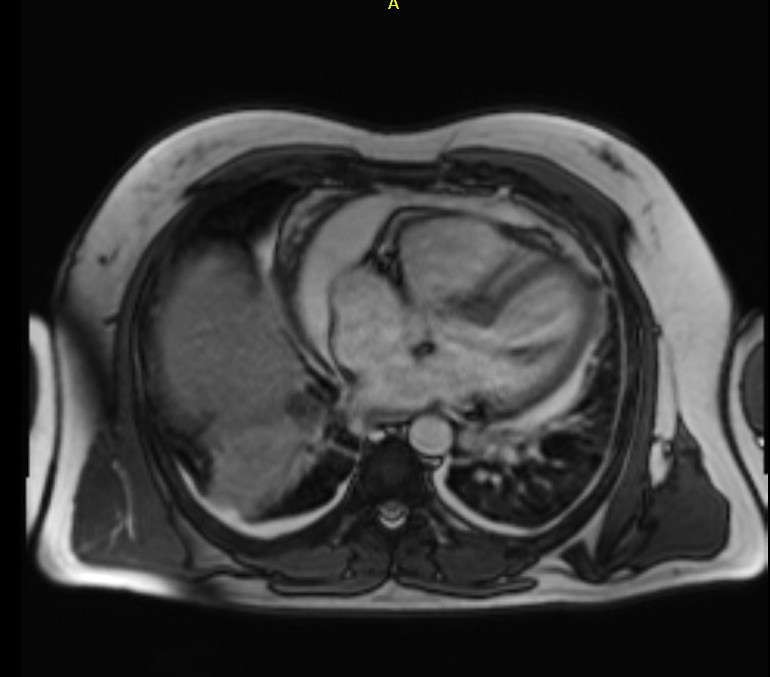


Figure S3:

Cardiac MRI after steroid treatment:
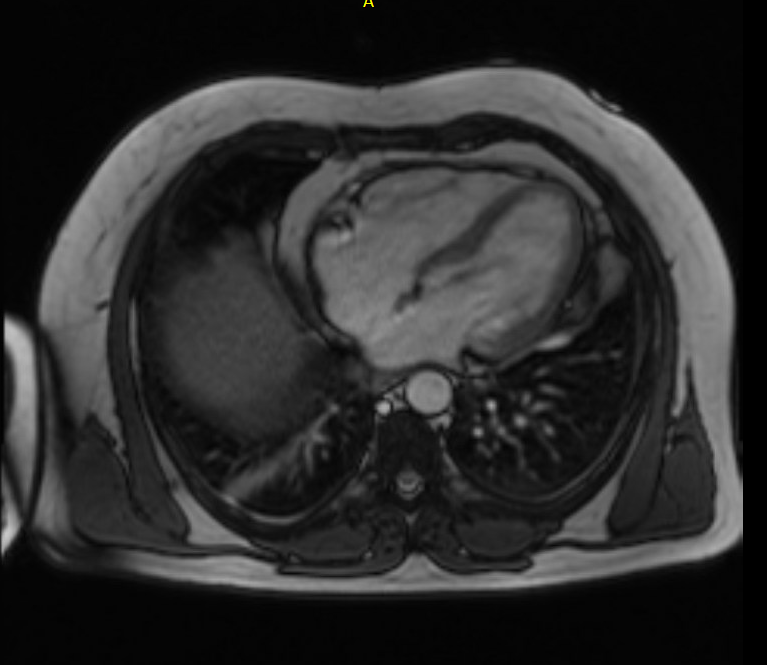


Figure. S4:

Inflammatory markers (WCC and CRP) trends with each admission:

|  | Admission | During admission | Discharge bloods |
| --- | --- | --- | --- |
| First presentation | WCC 11.2 × 10^9/L  CRP 227 mg/L | 8.6 × 10^9/L  212 mg/L | 6.7 × 10^9/L  55 mg/L |
| Second presentation | WCC. 17.5 × 10^9/L  CRP 216 mg/L | 14.7× 10^9/L  13 mg/L | 9.1 × 10^9/L  4 mg/L |
| Third presentation | WCC10.9 × 10^9/L  CRP 80 mg/L | 9.5 × 10^9/L  177 mg/L | 8.8 × 10^9/L  8 mg/L |
| Around pericardiectomy presentation | WCC 12.2 × 10^9/L  CRP 131 mg/L | 10.1× 10^9/L  15.1 mg/L | 24 × 10^9/L  5.2 mg/L |
